# Trend and factors associated with non-suppression of viral load among adolescents on ART in Tanzania: 2018–2021

**DOI:** 10.3389/frph.2024.1309740

**Published:** 2024-01-15

**Authors:** Andrewleon S. Quaker, Laura J. Shirima, Sia E. Msuya

**Affiliations:** ^1^ Institute of Public Health, Kilimanjaro Christian Medical University College, Moshi, Tanzania; ^2^ Regional Health Management Team, Regional Secretariat, Kilimanjaro, Tanzania

**Keywords:** HIV, suppression, Tanzania, adolescents, antiretroviral therapy (ART), viral load (VL)

## Abstract

**Background:**

Tanzania is one of the countries with a high burden of HIV. It has an estimated 1.4 million people living with HIV in 2021. Adolescents living with HIV on antiretroviral therapy (ART) have worse treatment adherence, viral suppression, and mortality rates compared to adults. This study aim was to determine the trend of non-suppression among adolescents on ART in Tanzania from 2018 to 2021 and latest associated predictors.

**Methodology:**

The study utilized data of adolescents (10–19 years) receiving ART in Tanzania mainland for the period of 2018–2021 from the National Care and Treatment Centers database. The primary outcome of interest was non-suppression of viral load, defined as a VL above 1,000 copies/ml. The study employed multivariable logistic regression models to identify factors associated with non-suppression of VL. STATA 15 statistical software was used to analyze the data.

**Results:**

Records of 65,942 adolescents present in the CTC database Tanzania were analyzed. Approximately more than half were female 38,544 (58.5%). The proportion of non-suppression was 34.5%, 23.3%, 12.1%, and 9.7% for the years 2018–2021, respectively. After adjusting for other factors, adolescents with a history of poor adherence to ART in the last six months had higher odds of non-suppression (OR = 1.95, 95% CI = 1.64, 2.31). Adolescents on second or third line ART regimens were almost two times more likely to be non-suppressed compared to those on first-line regimens (OR = 2.85, 95% CI = 2.52, 3.23). Girls had lower odds of non-suppression compared to boys (OR = 0.91, 95% CI = 0.84, 0.98), and similarly, patients attending hospitals had lower odds compared to those attending dispensaries (OR = 0.79, 95% CI = 0.72, 0.87).

**Conclusion:**

Being female, having good history of adherence over the last six months, and attending hospital level was significantly associated with lower levels of non-suppression, while being on second line ART or attending lower health facilities increased the odds of non-suppression. Efforts to enhance the quality and capacity of health services at lower-level facilities (dispensaries and health centers) should be prioritized, as well as promoting gender-sensitive approaches that take into account the unique needs and experiences of adolescent girls and boys are needed to improve VL suppression among this population.

## Introduction

HIV remains to be a great catastrophe for public health. In 2021 nearly 39.0 million individuals were living with the virus worldwide, of which 29.8 million (76.4%) people were accessing antiretroviral therapy (1). Sub-Sahara Africa remains to be the worst affected region across the world; which accounts for 74·0% of global HIV-related deaths and 64·9% of new HIV infections (2).

HIV continues to disproportionately impact young people from 10 to 24 years old especially adolescents from, 10 to 19 years, in particularly young women and vulnerable groups. The number of annual AIDS-related deaths among adolescents aged 10–19 has declined by 10% only from 2003 to 2020, while the decline was by 74% among children (3). Evidence indicates that adolescents living with HIV have significantly worse access to HIV testing, antiretroviral therapy coverage, and viral load suppression and further studies are required to understand how to tackle these challenges (4).

Achieving viral suppression and decreasing the number of HIV new infections is critical to achieving the HIV fast track *95*-*95*-*95* targets and eliminating AIDS as a public health threat by 2030 (5). The lack of data on viral load suppression among adolescents in sub-Saharan Africa is of particular of concern because the region is more hard-hit than other parts of the world with higher risks for mortality due to lack of treatment therefore their availability is crucial to ensure they are not left behind in the fight against the global HIV pandemic (6, 7).

The 2013 recommendation of WHO for viral load (VL) as a monitoring tool for patients on ARV was mainly aimed at resource-limited countries since many developed countries had access to VL testing for several years. Although reasons for high non-suppression rates have been explored among adult HIV-positive patients, and young age indicated as a predictor of non-suppression, few studies have focused on the factors that are associated with non-suppression among HIV-positive adolescents enrolled on ART (8). The proportion of adolescents with virological suppression after ART initiation varies from 27% to 89% globally (8). Most of the recent studies on viral load using WHO criteria have been conducted in Africa because developed countries have smaller number of HIV-positive adolescents and have set viral load cut points that are significantly smaller (50, 200, 400 copies per ml) than those set by the WHO of 1,000 copies per ml. Studies done in Asia and Middle East have relatively low prevalence's of non-suppression ranging from 17.9% to 23% (3, 9–11). In South African, a study showed that, among adolescents non-suppression is 33.3% in those aged 10–15 years (12). An early study conducted in Lesotho, Malawi, Mozambique, South Africa, Zambia and Zimbabwe showed that the proportion of non-suppressed adolescents among those with VL testing for the years 2005–2018 was 6% among older adolescents (13). In Uganda, non-suppression among the age groups (10–14 years) was 57% and was 27% in (15–19 years) (14). In Kenya a lower proportion of adolescents 12% aged 10–14 years who are VL non-suppressed (15).

Social demographic factors such as age, sex and caregiver support play a role in the social makeup of an individual and affecting his/her choices. Several studies have recognized age, gender, WHO stage, ART regimen, duration of treatment on ART, TB history, receiving services at lower health facilities as determinants of HIV VL non-suppression (16–20).

In Tanzania, HIV VL testing was introduced routinely in 2018. Two studies in Tanzania show the proportion of adolescents with viral non-suppression to be between 34.2% and 47.1% (21, 22). However, both studies were conducted using samples from regional data. The National AIDS Control Programme (NACP) produces annual reports on VL suppression. However, it does not report disaggregated virological non-suppression among the (10–19 years) adolescents receiving ART (23). Further, there is limited information on the trend of viral suppression among adolescents in Tanzania since the test was routinely introduced into care.

A more recent study that examined the trend of non-suppression from 2016 to 2018, reported that about one-third of young adolescents 10–14 years were not virally suppressed with the suppression rate remaining relatively static in Uganda while declining in Malawi and Zimbabwe (24). There is however scarcity of data on the trend of viral suppression among adolescents in Tanzania since viral load was introduced routinely in 2018.

This study aims determine the trends in HIV VL non-suppression among adolescents in Tanzania for the years 2018–2021 and the predictors of viral load non-suppression for the year 2021.

## Methods

### Study setting, design, and population

The study was a repeated cross-sectional study, using routinely collected program data extracted from the national Care and Treatment Center (CTC) electronic database of Tanzania mainland. The CTC is a government-run program providing comprehensive HIV care and treatment services, with over 400 sites located throughout Tanzania. All records of patients aged 10–19 who were on antiretroviral therapy (ART) for at least 6 months, attended CTC during the period of 2018–2021, and had at least one documented viral load were included in the study.

### Sample size

**Figure 1:**
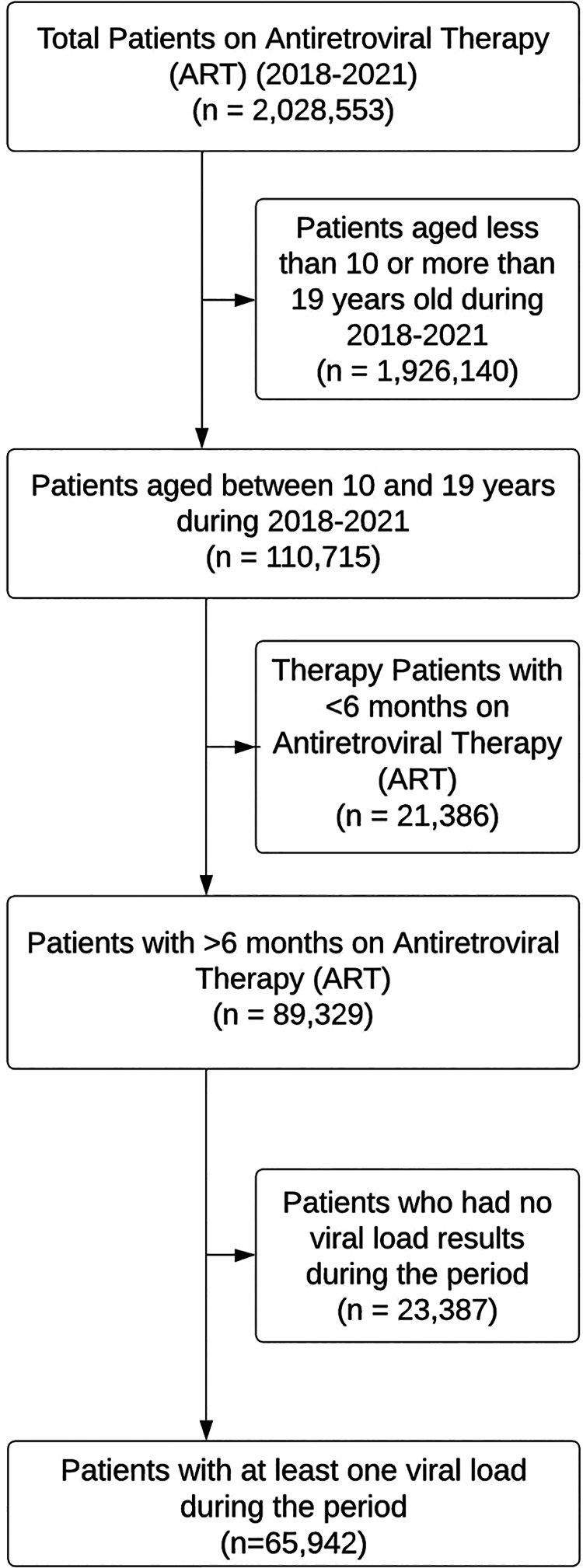
Flowchart of subjects selected for the study.

A total of 2,028,553 patients visited CTC clinics between 2018 and 2021, among which 110,715 were aged 10–19 years. Of these, 89,329 had been on ART for at least six months, but 23,387 had missing viral load results. Therefore, we analyzed a total of 65,942 patient records that had a documented viral load result during the period of 2018–2021 (Figure 1).

### Study variables

The outcome variable of interest was viral load suppression status, which was defined as a viral load of less than 1,000 copies/ml of blood. The viral non-suppression proportion was defined as the percentage of patients with viral load ≥1,000 copies/ml of blood among the total number of patients who had a viral load test. We also collected information on adolescents' characteristics, including age, sex, time on ART, WHO clinical stage, TB history, ART line, and reported adherence to ART within the last six months. ART adherence is routinely assessed by CTC staff during each patient visit, with “Good adherence” defined as pill utilization equal to or exceeding 95%. Using this criterion, we grouped all individuals who demonstrated good adherence in all visits over the six months before the viral load measurement as having “Good” Adherence to ART and those who had one or more visits where the adherence was poor as “Poor in the last six months”.

### Statistical analysis

Data from the CTC was analyzed yearly for estimating proportion of non-suppression using last viral load result for the specific year. Records of patients with missing viral load data were excluded from the study.

Univariate analysis was used to describe the socio-demographic and clinical characteristics of the study population. Proportion of adolescents with viral non-suppression was calculated for each year to show the overall trend. Subsequently, a chi-squared test for linear trend was performed to assess whether there is a overall linear trend over the years. To explore factors associated with viral non-suppression, we specifically examined the results of the year 2021. This decision was made to more clearly capture the most recent status, considering the ongoing improvements in healthcare that may have significantly influenced the variables under investigation over the years. Also the availability and completeness of data for the year 2021 ensured a robust analysis compared to earlier periods. We conducted bivariate and multivariable logistic regression analyses using Stata 15.0 (Stata Corporation, College Station, TX). We used univariate analyses to describe the socio-demographic and clinical characteristics of the study population. We used bivariate analyses to determine the strength of association between the independent variables and the outcome variable (viral suppression status). Crude odds ratios (cOR) and 95% confidence intervals (CI) were calculated. All variables that were significant in bivariate analysis were included in the multivariable analyses. We used multivariable logistic regression to identify factors independently associated with viral non-suppression. Adjusted odds ratios (aOR) and 95% confidence intervals were calculated. Cut-off for statistical significance was set at a *p*-value ≤ 0.05.

### Ethical considerations

The study was approved by the Kilimanjaro Christian Medical University College of Tumaini University Makumira ethical committee (No: PG 36/2021). Administrative approval was obtained from the Ministry of Health Community Development Elderly and Children (Ref. No. PA. 87/266/02-A/97). The study was conducted in accordance with the 1964 Declaration of Helsinki and its later amendments. As the study used routinely collected program data, informed consent was waived by the ethical committee. All patient data were de-identified prior to analysis to maintain confidentiality.

## Results

A total of 65,942 individuals aged 10–19 years who were present in the CTC database in Tanzania for the period of 2018–2021 were analyzed. The proportion of adolescents who had viral load results for four, three, two, and one year in the period 2018–2021 was 36.0%, 27.2%, 23.1%, and 13.7%, respectively (Supplementary Appendix I).

Characteristics of individuals based on the last viral load result for each of the years 2018–2021 are shown in Table 1. Approximately more than half were female 38,544 (58.5%), with a consistently higher proportion of younger adolescents (10–14 years).

**Table 1:** Population characteristics by year 2018–2021 (*N* = 65,942).

Characteristic	Year							
	2018		2019		2020		2021	
	*n*	%	*n*	%	*n*	%	*n*	%
Sex								
Male	13,468	44.5	16,623	43.8	16,880	43.6	15,154	44.1
Female	16,791	55.5	21,374	56.3	21,822	56.4	19,246	56.0
Age								
10–14 years	15,999	52.9	19,348	50.9	19,231	49.7	16,542	48.1
15–19 years	14,260	47.1	18,649	49.1	19,471	50.3	17,858	51.9
Mean(SD)	14.79 (1.98)		14.35 (1.49)		14.90 (2.00)		14.95 (1.74)	
Time on ART^a^								
Less than 1 year	638	2.1	1,003	2.6	1,039	2.7	743	2.2
1–3 years	10,876	36.0	12,182	32.1	10,874	28.1	8,598	25.0
4–6 years	8,410	27.8	11,013	29.0	11,407	29.5	9,765	28.4
7–10 years	7,769	25.7	9,662	25.5	10,228	26.5	9,982	29.1
11 years or more	2,550	8.4	4,108	10.8	5,127	13.3	5,277	15.4
Mean (SD)	5.82 (3.12)		5.82 (3.16)		5.97 (3.26)		6.41 (3.45)	
Adherence^b^								
Good	28,444	94	35,653	93.83	37,036	95.7	33,326	96.9
Poor in the last six months	1,815	6	2,344	6.17	1,666	4.3	1,074	3.1
ARV line^c^								
First line	27,576	91.5	33,648	88.8	35,734	92.4	32,519	94.6
Second or third line	2,577	8.6	4,261	11.2	2,938	7.6	1,868	5.4
TB status								
Negative	3,460	11.4	4,087	10.8	4,190	10.8	3,705	10.8
Positive	3,808	12.6	4,372	11.5	4,218	10.9	3,794	11.0
Unknown	22,991	76.0	29,538	77.7	30,294	78.3	26,901	78.2
WHO stage^d^								
Stage 1	3,503	11.9	5,103	14.0	5,631	15.5	5,381	16.7
Stage 2	5,053	17.1	6,030	16.5	5,658	15.6	5,092	15.8
Stage 3	16,317	55.3	19,616	53.7	19,213	53.0	16,915	52.5
Stage 4	4,645	15.7	5,794	15.9	5,763	15.9	4,805	14.9
Facility type^e^								
Dispensary	7,482	24.8	10,093	26.6	11,093	28.8	9,955	29.0
Health centre	8,751	29.0	11,890	31.4	12,451	32.3	11,530	33.6
Hospital	13,964	46.2	15,937	42.0	15,034	39.0	12,797	37.3
Zone^f^								
Central zone	2,404	8.0	3,412	9.0	3,785	9.8	3,617	10.6
Coastal zone	8,516	28.2	9,730	25.7	9,161	23.7	7,926	23.1
Lake zone	6,615	21.9	8,688	22.9	9,085	23.5	8,941	26.1
Northern zone	4,321	14.3	5,239	13.8	4,972	12.9	3,885	11.3
Southern highland zone	7,608	25.2	9,876	26.0	10,508	27.2	9,129	26.6
Western zone	733	2.4	975	2.6	1,077	2.8	784	2.3

Missing:

^a^ Time on ART 16, 29, 27, and 35.

^b^ Adherence 2,740, 4,002, 2,644 and 2,916.

^c^ ARV line 106, 88, 30 and 13.

^d^ WHO Stage 741, 1,454, 2,508 and 2,207.

^e^ Facility type 62, 77, 124 and 118.

^f^ Zone 62, 77, 124 and 118 for years 2018 to 2021 respectively.

### Trend of HIV VL non-suppression among adolescents

**Figure 2:**
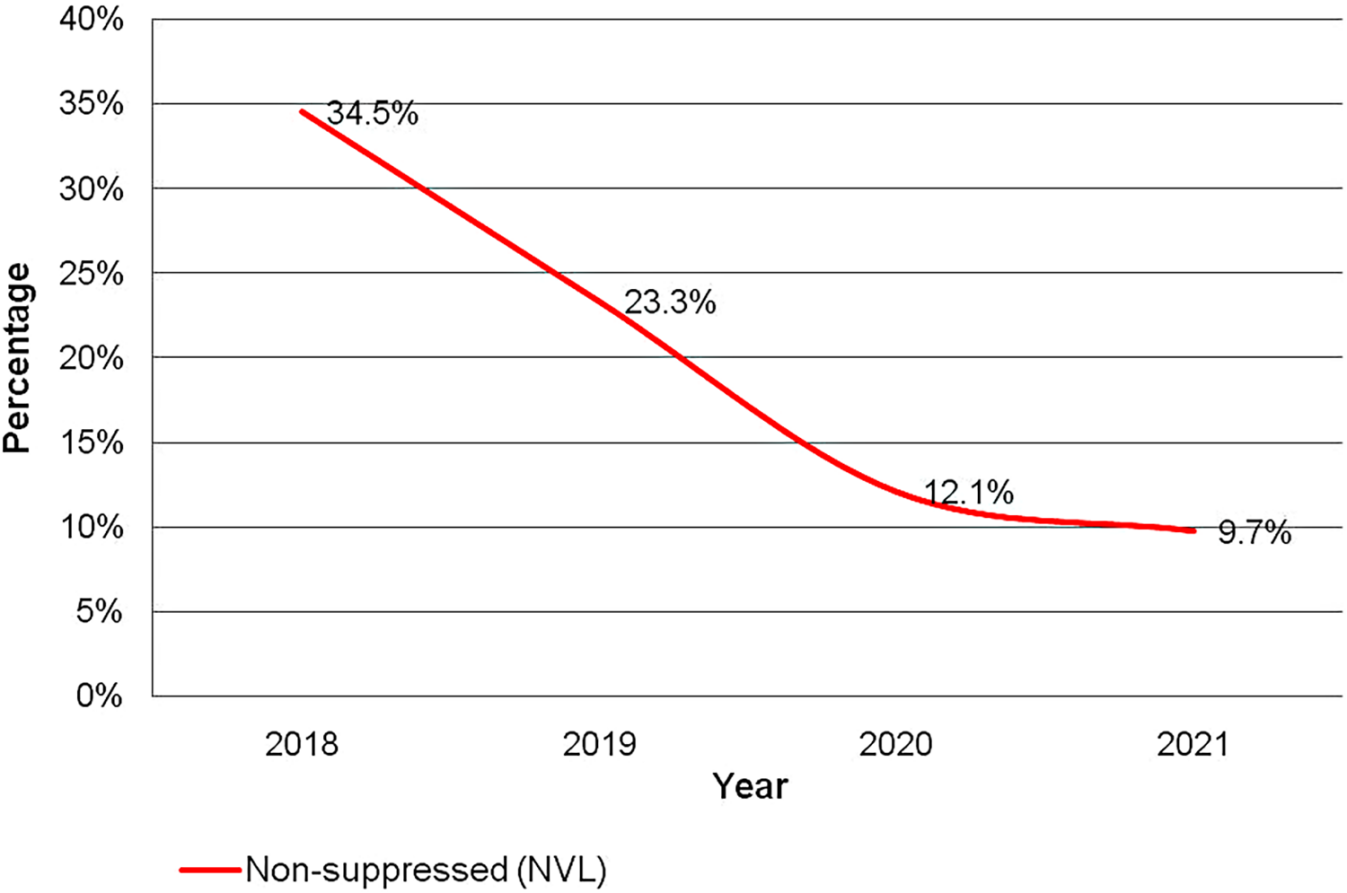
Trend of non-suppression of human immunodeficiency virus viral load among adolescents for the period of 2018–2021.

The proportion of adolescents who are VL non-suppressed has decreased from 2018 to 2021 from 34.5% to 9.7%, Figure 2. The observed threefold decrease in VL non-suppression, visually represented by a trend line, aligns with the statistical findings from chi-squared test for linear trend, which revealed a significant negative slope of approximately −8.56 (*p* = 0.03).

### VL non-suppression by selected demographic and clinical characteristics

Table 2 shows difference between groups on VL non-suppression by background characteristics. A higher proportion of male adolescents, adolescents with poor adherence to ART and on 2nd line ART treatment had higher occurrence of HIV VL non-suppression than others. The Southern Highland Zone had the highest proportion of adolescents who were VL non-suppressed, while the Lake zone had consistently achieved higher suppression compared to other zones. A higher proportion of adolescents using Dispensaries were VL non-suppressed than those accessing care at Health Centers, and Hospitals for ART services.

**Table 2:** Population characteristics of human immunodeficiency virus viral load non-suppressed adolescents according to last viral load results per the year 2021 (*N* = 34,400).

Characteristics	2021				
	Total	VL non-suppressed			
	*n*	*n*	%	*Χ* ^2^	*P*-Value
Sex				14.30	<0.001*
Male	15,154	1,579	10.4		
Female	19,246	1,771	9.2		
Age				0.22	0.638
10–14 years	16,542	1,598	9.7		
15–19 years	17,858	1,752	9.8		
Time on ART^a^					
Less than 1 year	743	55	7.4	13.73	0.008*
1–3 years	8,598	787	9.2		
4–6 years	9,765	952	9.8		
7–10 years	9,982	990	9.9		
11 years or more	5,277	564	10.7		
Adherence^b^				68.95	<0.001*
Good	33,326	3,166	9.5		
Poor in the last six months	1,074	184	17.1		
ARV line^c^				416.56	<0.001*
First line	32,519	2,910	9.0		
Second or third line	1,868	436	23.3		
History of TB				13.73	0.001*
No	3,705	381	10.3		
Yes	3,794	427	11.3		
Unknown	26,901	2,542	9.5		
WHO stage^d^				27.59	<0.001*
Stage 1	5,381	433	8.1		
Stage 2	5,092	495	9.7		
Stage 3	16,915	1,721	10.2		
Stage 4	4,805	523	10.9		
Facility type^e^				17.37	<0.001*
Dispensary	9,955	1,050	10.6		
Health centre	11,530	1,145	9.9		
Hospital	12,797	1,143	8.9		
Zone^f^				193.26	<0.001*
Central zone	3,617	276	7.6		
Coastal zone	7,926	1,010	12.7		
Lake zone	8,941	628	7.0		
Northern zone	3,885	346	8.9		
Southern highland zone	9,129	1,002	11.0		
Western zone	784	76	9.7		

* Statistically significant differences (*P* < 0.05) from the reference group.

Missing:

^a^ Time on ART 16, 29, 27, and 35.

^b^ Adherence 2,740, 4,002, 2,644 and 2,916.

^c^ ARV line 106, 88, 30 and 13.

^d^ WHO Stage 741, 1,454, 2,508 and 2,207.

^e^ Facility type 62, 77, 124 and 118.

^f^ Zone 62, 77, 124 and 118 for years 2018 to 2021 respectively.

### Factors associated with HIV viral non-suppression

The results of the bivariate and multivariable regression analyses for the last viral result for year 2021 (*N* = 34,400) are provided in Table 3. In bivariate analysis, sex, time on ART, ARV line, adherence to ART within the last six months, WHO stage, facility type, zone, were significantly associated with non-suppression of HIV viral load.

**Table 3:** Bivariate and multivariable logistic regression analysis of factors associated with non suppression of viral load among adolescents in Tanzania, for the year 2021 (*N* = 34,400).

Characteristics	Bivariate analysis			Multivariable analysis		
	cOR	95%CI	*P*-value	aOR	95%CI	*P*-value
Sex						
Male	1			1		
Female	0.88	(0.81,0.73)	<0.001*	0.91	(0.84,0.98)	0.014*
Age						
10–14 years	1			1		
15–19 years	1.02	(0.95,1.09)	0.638	0.97	(0.90,1.05)	0.472
Time on ART						
Less than 1 year	1			1		
1–3 years	1.26	(0.95,1.67)	0.111	0.23	(0.91,1.65)	0.172
4–6 years	1.35	(1.02,1.79)	0.037*	1.16	(0.86,1.57)	0.320
7–10 years	1.37	(1.04,1.83)	0.026*	1.13	(0.84,1.53)	0.416
11 years or more	1.50	(1.12,1.99)	0.006*	1.17	(0.86,1.59)	0.330
Adherence						
Good	1			1		
Poor in the last six months	1.97	(1.67,2.32)	<0.001*	1.95	(1.64,2.313)	<0.001*
ARV line						
First line	1			1		
Second or third line	3.10	(2.76,3.47)	<0.001*	2.85	(2.52,3.23)	<0.001*
TB Status						
Negative	1			1		
Positive	1.11	(0.96,1.28)	0.175	1.16	(1.00,1.35)	0.057
Unknown	0.91	(0.81,1.02)	0.106	1.02	(0.90,1.15)	0.780
WHO Stage						
Stage 1	1			1		
Stage 2	1.23	(1.08,1.40)	0.003*	1.48	(1.40,1.57)	<0.001*
Stage 3	1.29	(1.16,1.45)	<0.001*	1.52	(1.45,1.59)	<0.001*
Stage 4	1.40	(1.22,1.60)	<0.001*	1.58	(1.49,1.57)	<0.001*
Facility type						
Dispensary	1			1		
Health centre	0.94	(0.86,1.02)	0.089	0.95	(0.86,1.04)	0.285
Hospital	0.83	(0.76,0.91)	<0.001*	0.79	(0.72,0.87)	<0.001*
Zone						
Central zone	1			1		
Coastal zone	1.77	(1.54,2.03)	<0.001*	1.51	(1.13,1.74)	<0.001*
Lake zone	0.91	(0.79,1.06)	0.234	0.87	(0.75,1.01)	0.063
Northern zone	1.18	(1.00,1.40)	0.046*	1.20	(1.02,1.43)	0.030*
Southern highland zone	1.49	(1.30,1.72)	<0.001*	1.45	(1.25,1.67)	0.005*
Western zone	1.30	(1.00,1.70)	0.054	1.19	(0.90,1.57)	0.213

cOR, crude odds ratio; aOR, adjusted odds ratio.

* Statistically significant differences (*P* < 0.05) from the reference group.

Findings from the multivariable analysis indicate that girls had 9% lower odds of being non-suppressed compared to boys (AOR = 0.91; 95% CI = 0.84, 0.98), also those with a history of having poor adherence to ART within the last six months had 95% higher chances of being non-suppressed (AOR = 1.95; 95% CI = 1.64, 2.31). Patients on second or third line ART regimen were almost 2 times more likely to be non-suppressed (AOR = 2.85; 95% CI = 2.52, 3.23). Advanced WHO stages were significantly associated with higher odds of non-suppression when compared to those with stage one. Regarding facilities patients attending hospital level were significantly at 0.21 lower odds for non-suppression compared to those attending dispensaries (AOR = 0.79; 95% CI = 0.72, 0.87). Patients in the Coastal, Northern, and Southern Highland zones had significantly higher odds of being non-suppressed when compared to the Central zone (AOR = 1.51, 95% CI = 1.13, 1.74; AOR = 1.2, 95% CI = 1.02, 1.43; and AOR = 1.45, 95% CI = 1.25, 1.67), respectively.

## Discussion

The study results showed that the proportion of adolescents who were HIV VL non-suppressed was 34.5%, 23.3%, 12.1% and 9.7% for the years 2018–2021 respectively. The trend exhibited a sharp decline in the first 3 years followed by a lesser change in the last year. The overall decrease in non-suppression is greater than 3-fold since 2018. Being male, having a history of poor adherence to ART within last six months, on second or third line ART, advanced WHO stage, attending dispensary level facility, in Coastal, Northern and Southern Highland zones were significantly associated with increased odds of Non-suppression of Viral load.

There are a relatively high proportion of adolescents who were viral load non-suppressed in the first two years of the study. This initial proportion is comparable with that reported in the WHO multi-country study on VL non-suppression among children and adolescents for the years 2016–2018, whereby, about one third were not suppressed (24). The last two years of the study showed comparatively lower proportions of VL non-suppression although relatively higher than that of 6% reported among older adolescents in Malawi, Mozambique, South Africa Zambia and Zimbabwe between 2005 and 2018 (13). This could be explained by the smaller sample of adolescents in the second study whose majority of participants were adults. The rapid decrease in the proportion of HIV VL non-suppressed can be attributed to access to viral load testing and improvement in care services as illustrated in China and Haiti (25, 26). Similarly, these findings are considerably lower than those reported in studies using regional data from Tanzania (21, 22).

The decreasing trend in VL non-suppression rates over time among adolescents living with HIV who are on antiretroviral therapy (ART) has several implications. Firstly, it indicates that the current ART regimens are more effective in suppressing the virus and reducing the risk of HIV transmission. Secondly, it suggests that efforts to improve adherence to ART and increase access to treatment in terms of infrastructure service and supplies are bearing fruit, meaning that healthcare providers are doing a better job of ensuring patients are able to adhere to their treatment plans. Thirdly it highlights the importance of ongoing monitoring and evaluation of ART programs. Understanding what those changes were and why they were effective is important for improving the effectiveness of ART programs in the future.”

The recent 2021 proportion of adolescents (9.7%) who were HIV VL non-suppressed (90.3% VL suppression) is relatively on track for the Joint United Nations Programme on HIV/AIDS program's goal of achieving 95% viral suppression by 2025. The variation in the proportion of adolescents who were VL non-suppressed seen between years, type of facilities and zones could be due to the presence of external factors such as the improvement in HIV care over time (years). Tanzania commission for AIDS (TACAIDS) and the National AIDS Control Program (NACP) have developed and started implementing strategic plans which targets to expand ART coverage to 95% for all PLHIV by 2025 and reduce new infections to only 15,000 annually by 2023 (27, 28). More recently the Ministry of Health has launched the National Accelerated Action and Investment Agenda for Adolescent Health and Wellbeing (NAIA-AHW) which recognized the gaps in adolescent care and intends to operationalize and streamline interventions at the national and sub-national level including access to Healthcare and HIV prevention (29).

While many studies focus on the 12 months proportions of VL non-suppression, our study looked at yearly proportions over a period of 4 years 2018–2021. The high burden observed over the first 2 years, reflects the fact that reporting aggregated data for the 15+ to some extent masked and failed to report this problem among adolescents. The decreasing trend in the proportion of seen over the years and decrease in differences between the proportions by age and sex groups highlights that, VL non-suppression can be substantially reduced in Tanzania to <5%, putting the 2025 Goal within reach.

In this study, we found that males where significantly at higher odds of being virally non-suppressed. This is similar to studies done in other countries were males were at higher odds of being non-suppressed (30, 31). Males tend to be more rebellious and have poorer adherence to medication. Sex difference in viral suppression rate should be studied further.

Our study also investigated the association between duration of antiretroviral therapy (ART) and non-viral load suppression (NVL) among adolescents living with HIV. Bivariate analysis showed that adolescents on ART for 1–3 years, 4–6 years, 7–10 years, and 11 years or more had a higher odds ratio (OR) of having NVL compared to those on ART for less than 1 year. However, the multivariable analysis adjusted for other factors showed that only adolescents on ART for 4–6 years had a slightly increased adjusted odds ratio (aOR) of having NVL, but this association was not statistically significant (*p* = 0.32). This finding is consistent with previous studies that have reported a higher risk of NVL among those on ART for a longer duration (32, 33).

In contrast to several previous studies, our findings suggest that there is no significant difference in the risk of viral load non-suppression between younger (10–14 years) and older (15–19 years) age groups. This finding is consistent with a recent study conducted in Kenya that also reported no significant difference in viral suppression between these age groups (34). It is possible that this difference has decreased over time due to improvements in health service delivery, which have created greater equity in treatment for all age groups. Further research is needed to explore this finding in more detail and to better understand the factors that contribute to NVL in adolescents.

Several other studies have also reported a strong association between poor adherence and non-viral load suppression, which is consistent with the findings of this study. Poor adherence to ART has been recognized as a significant contributor to treatment failure and the development of drug resistance in HIV-positive patients. Adherence can be influenced by a range of factors, including medication side effects, patient education, and counseling, as well as access to medications (35). However, the impact of poor adherence on viral load suppression is complex and multifaceted, as other factors such as treatment regimen complexity, drug toxicity, and patient co morbidities can also contribute to poor suppression (36). The measurement of Adherence has been a contentious issue with different approaches, including self-reported adherence, pill counts, and electronic monitoring devices utilized in numerous studies (37). In this study poor adherence was defined as poor adherence within the last six months, this definition is consistent with existing measurements routinely used in the clinic and the current practice of multi-month prescription for stable patients.

The study found that advanced WHO stages (stage 3&4) were significantly associated with higher odds of VL non-suppression when compared to those with stage one, which is consistent with previous studies conducted in Kenya and Ethiopia (18, 34, 38). These findings suggest that being in the advanced WHO stages of HIV is an important risk factor for non-suppression probably due to a variety of psychosocial economic and biological factors that resulted in the patient reaching such stage. There is dire need to develop targeted interventions that address the unique needs of individuals in the later stages of HIV in order to improve viral suppression rates.

Adolescents attending lower-level facilities (dispensary) were at higher odds of VL non suppression than those attending hospitals, a finding also shown in the Ethiopian study (18, 19). This could be a reflection of poor provider skills that may translate into lower quality of service at lower facilities and also lower socioeconomic status of patients who have poorer access to higher level facilities. Further studies on quality of service and determinants of facility choice could be undertaken. Training of staff in to improve health service in dispensary and health center could be of benefit.

The study showed that there is zonal variation in the experience of VL non-suppression among adolescents in Tanzania. The Western, Southern highlands, Southern, and Coastal zones have significantly higher odds of VL non-suppression. This variation may reflect health system utilization and service provision across regions. Consequently, since viral load services are provided at specific centers Zonal Referral Hospital utilizing a hub and spoke model, road infrastructure, accessibility and sample transport may play a role in terms of availability and quality of results. Moreover, these findings have policy implications, as interventions aimed to reduce NVL will have to be more holistic in their approach, going beyond the descriptive zonal variables.

### Study limitation and strengths

This study utilized data from the national CTC database and included all records which means the findings are generalizable to adolescents living with HIV in Tanzania. Also, the study had enough power to conclude on the factors associated with HIV VL non-suppression among adolescents aged 10–19 years in Tanzania.

The nature of the data showed that there are factors operating beyond the individual level, i.e., at the facility level, zonal level. The rapid reduction in non-suppressed adolescents has a policy implication as it reflects existing programs and intervention by the government and other stakeholders have been effective and sheds a little more light on underserved areas in Tanzania.

The study does have some limitations which need to be considered when interpreting the results. Firstly, using secondary data, the study was limited to evaluating the variables as found in the database. Data was collected routinely from care and treatment centers in Tanzania therefore lack of some socio-demographic variables such as education and economic status does not capture all relevant information for determining viral suppression such as the impact of medication side effects or co-morbidities on viral suppression rates.

Secondly, the outcome is based on the last viral load within a period of one year and not repeated measures such as utilized for weighted analysis or virological failure. Despite that, last viral loads for a specific period has been shown to truly reflect the state of patients and has been recommended as a suitable measure for last viral load suppression to monitor national HIV Strategy (39).

Thirdly, our study was cross-sectional in nature, given the current developments in HIV care and treatment more focused cohort studies are needed to better understand the factors that contribute to non-suppression of viral load over time among adolescents living with HIV.

Lastly, our study did not include qualitative data on patients' experiences with ART, which could have provided important insights into the factors that influence adherence and viral suppression among adolescents living with HIV. In future research, it may be beneficial to incorporate qualitative data in addition to quantitative data to gain a more comprehensive understanding of the complex factors that impact HIV treatment outcomes among adolescents living with HIV.

## Conclusions and recommendations

This was the first study to examine the trend of non-suppression of HIV viral load among adolescents since the introduction of routine viral load monitoring in Tanzania and the recent predictors of non-suppression. Our study found a significant decrease in proportions of adolescents with non-suppressed viral load. The study also confirms adherence as the single most important factor associated with achievement of viral suppression. Good adherence was key to viral suppression regardless of age, time on ART, gender or ART line. The study also confirms that boys are at higher risk of being non-suppressed. This finding is concerning and should be further investigated to discover reasons and interventions that could address this disparity among adolescents on ART. Lower level facilities need to be strengthened to ensure that any barriers preventing those attending from achieving suppression similar to those attending higher level facilities are identified and addressed. Further studies (qualitative or mixed methods) that explore the social, cultural, and economic factors that affect adherence and viral suppression among adolescents could provide valuable insights into strategies to improve outcomes.

## Data Availability

The datasets presented in this article are not readily available because it contains protected health information. Request to access data should be directed to the Ministry of Health, after approval from National Institute of Medical Research (NIMR) and Tanzania Commission for Science and Technology (COSTECH).

## References

[1] Joint United Nations Programme on HIV/AIDS (UNAIDS). (2023). “*Global HIV & AIDS Statistics Fact Sheet*.” Unaids. 2023. Available at: https://www.unaids.org/en/resources/fact-sheet 12349391

[2] Institute for Health Metrics and Evaluation (IHME). (2021). “*HIV Incidence & Mortality.*” Seattle, WA: IHME, University of Washington. 2021. Available at: https://www.healthdata.org/research-analysis/diseases-injuries/hiv-aids

[3] UNICEF. (2021). “*HIV Statistics—Global and Regional Trends—UNICEF DATA.*” *National Agency for Control of AIDS*. Available at: https://data.unicef.org/topic/hivaids/global-regional-trends/

[4] Armstrong A Nagata JM Vicari M Irvine C Cluver L Sohn AH A global research agenda for adolescents living with HIV. JAIDS J Acquir Immune Defic Syndr. (2018) 78(1):S16–21. 10.1097/QAI.0000000000001744 29994915 PMC6075888

[5] UNAIDS. (2015). “*Understanding Fast-Track Targets. Accelerating Action to End the AIDS Epidemic by 2030*.” *Unaids*. Available at: https://www.unaids.org/sites/default/files/media_asset/201506_JC2743_Understanding_FastTrack_en.pdf

[6] GoTZ, and UNICEF. (2018). “HIV and AIDS Budget Brief 2018 Tanzania.” Dar Es Salaam. Available at: http://www.nacp.go.tz

[7] UNAIDS. (2019). “*Seizing the Moment: Tackling Entrenched Inequalities to End Epidemics*.” *GLOBAL AIDS UPDATE | 2020*. Vol. 72. Geneva.

[8] Ferrand RA Briggs D Ferguson J Penazzato M Armstrong A MacPherson P Viral suppression in adolescents on antiretroviral treatment: review of the literature and critical appraisal of methodological challenges. Trop Med Int Health. (2016) 21(3):325–33. 10.1111/tmi.12656 26681359 PMC4776345

[9] Bartlett AW Sudjaritruk T Mohamed TJ Anugulruengkit S Kumarasamy N Phongsamart W Identification, management, and outcomes of combination antiretroviral treatment failure in adolescents with perinatal human immunodeficiency virus infection in Asia. Clin Infect Dis. (2021) 73(7):e1919–26. 10.1093/cid/ciaa872 32589711 PMC8492217

[10] Chhim K Mburu G Tuot S Sopha R Khol V Chhoun P Factors associated with viral non-suppression among adolescents living with HIV in Cambodia: a cross-sectional study. AIDS Res Ther. (2018) 15(1):1–10. 10.1186/s12981-018-0205-z 30445984 PMC6240223

[11] Mu W Bartlett AW Bunupuradah T Chokephaibulkit K Kumarasamy N Ly PS Early and late virologic failure after virologic suppression in HIV-infected Asian children and adolescents. JAIDS J Acquir Immune Defic Syndr. (2019) 80(3):308–15. 10.1097/QAI.0000000000001921 30531299 PMC6952284

[12] Van Liere GAFS Lilian R Dunlop J Tait C Rees K Mabitsi M High rate of loss to follow-up and virological non-suppression in HIV-infected children on antiretroviral therapy highlights the need to improve quality of care in South Africa. Epidemiol Infect. (2021) 149:e88. 10.1017/S0950268821000637 33745490 PMC8080219

[13] Haas AD Radin E Hakim AJ Jahn A Philip NM Jonnalagadda S Prevalence of nonsuppressed viral load and associated factors among HIV-positive adults receiving antiretroviral therapy in Eswatini, Lesotho, Malawi, Zambia and Zimbabwe (2015 to 2017): results from population-based nationally representative surveys. J Int AIDS Soc. (2020) 23(11):1–12. 10.1002/jia2.25631 PMC7680921 33225559

[14] Bulage L Ssewanyana I Nankabirwa V Nsubuga F Kihembo C Pande G Factors associated with virological non-suppression among HIV-positive patients on antiretroviral therapy in Uganda, August 2014–July 2015. BMC Infect Dis. (2017) 17(1):326. 10.1186/s12879-017-2428-3 28468608 PMC5415758

[15] Nabukeera S Kagaayi J Makumbi FE Mugerwa H Matovu JKB. Factors associated with virological non-suppression among HIV-positive children receiving antiretroviral therapy at the joint clinical research centre in Lubowa, Kampala Uganda. PLoS One. (2021) 16(1 January):1–12. 10.1371/journal.pone.0246140 PMC7840004 33503074

[16] Afrane AKA Goka BQ Renner L Yawson AE Alhassan Y Owiafe SN. HIV virological non-suppression and its associated factors amongst children on antiretroviral therapy at a major paediatric treatment centre in Southern Ghana: a cross-sectional study. BMC Infect Dis. (2021) 21(731):1–11. 10.1186/s12879-021-06459-z 34340689 PMC8330060

[17] Costenaro P Penazzato M Lundin R Rossi G Massavon W Patel D Predictors of treatment failure in HIV-positive children receiving combination antiretroviral therapy: cohort data from Mozambique and Uganda. J Pediatric Infect Dis Soc. (2015) 4(1):39–48. 10.1093/jpids/piu032 26407356

[18] Desta AA Woldearegay TW Futwi N Gebrehiwot GT Gebru GG Berhe AA HIV virological non-suppression and factors associated with non-suppression among adolescents and adults on antiretroviral therapy in Northern Ethiopia: a retrospective study. BMC Infect Dis. (2020) 20(1):1–10. 10.1186/S12879-019-4732-6 PMC6941313 31898535

[19] Haghighat R Toska E Bungane N Cluver L. The HIV care cascade for adolescents initiated on antiretroviral therapy in a health district of South Africa: a retrospective cohort study. BMC Infect Dis. (2021) 21:1. 10.1186/s12879-020-05742-9 33435861 PMC7805141

[20] UNICEF. (2020). “HIV and AIDS.” Dar Es Salaam: UNICEF. Available at: https://www.unicef.org/tanzania/media/2436/file/HIVProgramme Fact Sheet.pdf

[21] Bitwale NZ Mnzava DP Kimaro FD Jacob T Mpondo BCTT Jumanne S. Prevalence and factors associated with virological treatment failure among children and adolescents on antiretroviral therapy attending HIV/AIDS care and treatment clinics in Dodoma Municipality, Central Tanzania. J Pediatric Infect Dis Soc. (2021) 10(2):131–40. 10.1093/jpids/piaa030 32463083

[22] Sinai I Bowsky S Cantelmo C Mbuya-Brown R Panjshiri Y Balampama M. Adolescent HIV in Tanzania: Factors Affecting Viral Load Suppression and the Transition to Adult Care. Washington, DC: (Palandium) Health Policy Plus (2019).

[23] TACAIDS and ZAC. (2018). “Tanzania HIV Impact Survey (THIS) 2016-2017: Final Report.” *Dar Es Salaam*. Available at: https://phia.icap.columbia.edu/wp-content/uploads/2019/06/FINAL_THIS-2016-2017_Final-Report__06.21.19_for-web_TS.pdf

[24] UNICEF. (2021). “Understanding and Improving Viral Load Suppression in Children with HIV in Eastern and Southern Africa.” *UNICEF*. Nairobi. Available at: https://www.unicef.org/esa/reports/understanding-and-improving-vls

[25] Liu P Liao L Xu W Yan J Zuo Z Leng X Adherence, virological outcome, and drug resistance in Chinese HIV patients receiving first-line antiretroviral therapy from 2011 to 2015. Medicine (United States). (2018) 97(50):1–7. 10.1097/MD.0000000000013555 PMC6320000 30558015

[26] Wang Y Barnhart S Francois K Robin E Kalou M Perrin G Expanded access to viral load testing and use of second line regimens in Haiti: time trends from 2010 to 2017. BMC Infect Dis. (2020) 20(1):1–13. 10.1186/s12879-020-04978-9 PMC7160963 32299389

[27] TACAIDS. *Tanzania National Multisectorial Strategic Framework for HIV and AIDS 2018/19 to 2022/23*. Vol. 0 (2018).

[28] MoHCDEC. (2017). National AIDS Control Programme (HSHSP IV) 2017–2022.Available at: http://www.mcdgc.go.tz/

[29] MoHCDEC. National Accelerated Action and Investment Agenda for Adolescent Health and Wellbeing (Naia-Ahw) 2021/22–2024/25 (2021).

[30] Njuguna I Neary J Mburu C Black D Beima-Sofie K Wagner AD Clinic-level and individual-level factors that influence HIV viral suppression in adolescents and young adults: a national survey in Kenya. AIDS. (2020) 34(7):1065–74. 10.1097/QAD.0000000000002538 32287060 PMC7274775

[31] Diress G Dagne S Alemnew B Adane S Addisu A. Viral load suppression after enhanced adherence counseling and its predictors among high viral load HIV seropositive people in North Wollo Zone public hospitals, Northeast Ethiopia, 2019: retrospective cohort study. AIDS Res Treat. (2020) 2020:8909232. 10.1155/2020/8909232 32373359 PMC7191360

[32] Boerma RS Sonia Boender T Bussink AP Calis JCJ Bertagnolio S Rinke de Wit TF Suboptimal viral suppression rates among HIV-infected children in low- and middle-income countries: a meta-analysis. Clin Infect Dis. (2016) 63(12):1645–54. 10.1093/cid/ciw645 27660236

[33] Sher R Dlamini S Muloiwa R. Patterns of detectable viral load in a cohort of HIV-positive adolescents on antiretroviral therapy in South Africa. J Int AIDS Soc. (2020) 23(3):1–6. 10.1002/jia2.25474 PMC7076279 32180367

[34] Mwangi A van Wyk B. Factors associated with viral suppression among adolescents on antiretroviral therapy in Homa Bay County, Kenya: a retrospective cross-sectional study. HIV/AIDS—Research and Palliative Care. (2021) 13:1111–18. 10.2147/HIV.S345731 34992469 PMC8713714

[35] Kvarnström K Westerholm A Airaksinen M Liira H. Factors contributing to medication adherence in patients with a chronic condition: a scoping review of qualitative research. Pharmaceutics. (2021) 13(7):1100. 10.3390/pharmaceutics13071100 34371791 PMC8309154

[36] Byrd KK Hou JG Hazen R Kirkham H Suzuki S Clay PG Antiretroviral adherence level necessary for HIV viral suppression using real-world data. JAIDS J Acquir Immune Defic Syndr. (2019) 82(3):245–51. 10.1097/QAI.0000000000002142 31343455 PMC6854523

[37] Hodes R Cluver L Toska E Vale B. Pesky metrics: the challenges of measuring ART adherence among HIV-positive adolescents in South Africa. Crit Public Health. (2020) 30(2):179–90. 10.1080/09581596.2018.1550253

[38] Kim MH Mazenga AC Yu X Ahmed S Paul ME Kazembe PN High self-reported non-adherence to antiretroviral therapy amongst adolescents living with HIV in Malawi: barriers and associated factors. J Int AIDS Soc. (2017) 20(1):21437. 10.7448/IAS.20.1.21437 28406275 PMC5515061

[39] Xia Q Wiewel EW Braunstein SL Kersanske LS Torian LV. Comparison of indicators measuring the proportion of human immunodeficiency virus-infected persons with a suppressed viral load. Ann Epidemiol. (2015) 25(4):226–30. 10.1016/j.annepidem.2015.01.014 25727312

