# Supplementary material for: Trend and factors associated with non-suppression of viral load among adolescents on ART in Tanzania: 2018–2021
Source: Front Reprod Health. 2024 Jan 15;6:1309740. doi: 10.3389/frph.2024.1309740 (PMC10823012; doi:10.3389/frph.2024.1309740)
Supplement: Supplementary file 1 [file Table1.docx]

## Appendix I: Last viral load per year results frequencies for the period of 2018/21

**Table A: Distribution of last viral load per year for subject during the period of 2018/21 (N=65,942)**

| Number of last viral load per year | Number of Subjects  n | Percentage |
| --- | --- | --- |
| One year result | 23,767 | 36.04 |
| Two years results | 17,940 | 27.21 |
| Three year results | 15,229 | 23.09 |
| Four year results | 9,006 | 13.66 |
| **Total number of Subjects** | **65,942** | **100** |
